# Theories of Aging and the Prevalence of Alzheimer's Disease

**DOI:** 10.1155/2019/9171424

**Published:** 2019-06-16

**Authors:** Kaynara Trevisan, Renata Cristina-Pereira, Danyelle Silva-Amaral, Tales Alexandre Aversi-Ferreira

**Affiliations:** ^1^Laboratory of Physical Anthropology and Biomathematics, Department of Anatomy, Institute of Biomedical Science, Federal University of Alfenas, Alfenas, Brazil; ^2^Department of Physiology, School of Medicine and Pharmaceutical Sciences, System Emotional Science, University of Toyama, Toyama, Japan

## Abstract

**Objective:**

Aging and AD are associated in some way, then it is reasonable to ask whether or not it is possible to age without AD inexorably appearing at any moment, depending on the period of life. Therefore, the goal of this review is to verify, in light of some aging theories, the prevalence of AD.

**Methods:**

For the purpose of this manuscript, the indexers* Alzheimer*,* aging*,* Alzheimer*, and* aging *were considered;* theories of aging* were researched. The research was conducted using PubMed, Medline, Scopus, Elsevier, and Google Scholar.

**Results:**

The most common subjects in the papers analyzed for this manuscript were aging and Alzheimer's disease. The association between Alzheimer and theories of aging seems inconclusive.

**Conclusions:**

Accordingly, the general idea is that AD is associated with aging in such a way that almost all people will present this disease; however, it is plausible to consider that the increase in life expectancy will generate a high prevalence of AD. In a general sense, it seems that the theories of aging explain the origin of AD under superlative and catastrophic considerations and use more biomolecular data than social or behavioral data as the bases of analysis, which may be the problem.

## 1. Introduction

Alzheimer's disease (AD) was first studied and discovered by the German Psychiatrist Alois Alzheimer [1] in 1906 as the main cause of senile dementia. There is no way to definitively diagnose it during life [2] and, until now, presented an unknown, or multifactorial etiology [3–5]. AD is likely associated with environmental and genetic factors [6, 7], which generate a heterogeneous neurodegenerative disease [8], and research conducted on non-human subjects has demonstrated little potential for preclinical use [9].

Several papers have associated AD with aging [10–13], obviously because AD generally (in about 90% of cases) [14] affects individuals from the age of 65 and its prevalence doubles each 5 years, generating a time-dependent exponential increase [15].

In terms of the effects of this disease, AD is strongly associated with neurodegeneration and decreased cognition [1, 16] including language capabilities, praxis, loss of memory [8, 17] with loss of ability to recognize faces and recall names [18–20], loss of judgement and emotional stability [3, 21, 22], personality alterations [10], progressive and increased loss of neurons with presence of senile plaques, neurofibrillary tangles [10, 23], widespread neuronal network destruction [24, 25], brain [26], and evident hippocampal atrophy [27, 28]; however, several factors are associated with normal aging.

The association of AD with aging seems, at least according to some papers [14], to indicate that the majority of elderly people are subject to a high probability of having it and, considering the prevalence of other dementia, almost all elderly people have the potential, but not necessarily, to present some type of elderly disease during the third age, including Parkinson disease [29]. The above information was based on the reasoning of the theories of aging [30].

Accordingly, with the increasing average life of the population, dementia in general and, particularly, Alzheimer's, will be a public health issue [31] or, at least, a social concern [21, 32, 33]. The problem became more concerning after a recent study showed that senile dementia could potentially generate other pathologies caused by neuronal degeneration, for instance bipolar disorder [34].

Many theories were proposed in attempt to explain the process of aging and, generally, they can be divided into two groups: stochastic and non-stochastic [35]. The stochastic group is associated with molecular degradation and the actions of free radicals [36] generating a cumulative effect [37] on the cell's components [38].

The non-stochastic theories of aging are associated with the degradation of genes during the aging process, considering environmental influence [21].

Indeed, and independent of aging theories, aging and AD are associated in some way [3]. Therefore, it is reasonable to ask whether or not it is possible to age without AD inexorably appearing at any moment, depending on the period of life.

Therefore, the goal of this review is to assemble some commentary, in light of some aging theories, on the prevalence of AD.

## 2. Materials and Methods

For the purpose of this manuscript, 125 papers and a few books considering the indexers* aging*,* Alzheimer disease*,* Alzheimer *and* aging*,* theories of aging* were researched and analyzed. The research was conducted using articles from PubMed, Medline, Scopus, Elsevier, and Google Scholar under the languages of English, Spanish, and Portuguese.

The exclusion criteria for the papers eliminated those that were out of the scope this article, i.e., after establishing the goal; we sought papers that fit with the objective of associating DA with aging. As for books, only those most representative of the theme were selected, especially The Biology of Aging [39], a book we considered a classic regarding aging theories.

A lot of papers on DA, aging, and the association of both were found on the data bases but only those from best scientific journals, and some directly linked to the goal of this article, were chosen.

In order to relate the number of papers and their subjects in general terms, the qui-square test using the program StatPlus:mac AnalystSoft Inc. 2018 was applied. However, in order to improve statistical analysis, a Kruskal-Wallis ANOVA was performed using the same program.

The qui-square is considered robust for small samples and non-parametric analysis. The expected data were obtained by dividing the number of papers (totaling 100%) by the number of subjects, considering that these subjects have the same probability of being studied. For the Kruskal-Wallis ANOVA, the samples were considered independent.

Another analysis was performed associating the theories of aging and its probability of generating AD, in terms of basic probability. The theories, i.e., each one, were considered to have the same probability of generating AD, according to a qualitative analysis (see discussion below). If the theory of aging indicated at least one possibility of generating AD, it was considered positive with regard to generating AD. If the data from that theory did not present any possibility of generating AD, it was considered negative. For instance, if the theory of somatic mutations shows any possibility of generating AD, it was included in the calculation of probability; i.e., it was considered as positive. On the contrary, if an aspect of the aging theory was not linked to DA, it was removed from the calculation, i.e., was considered negative.

## 3. Results

**Figure 1:**
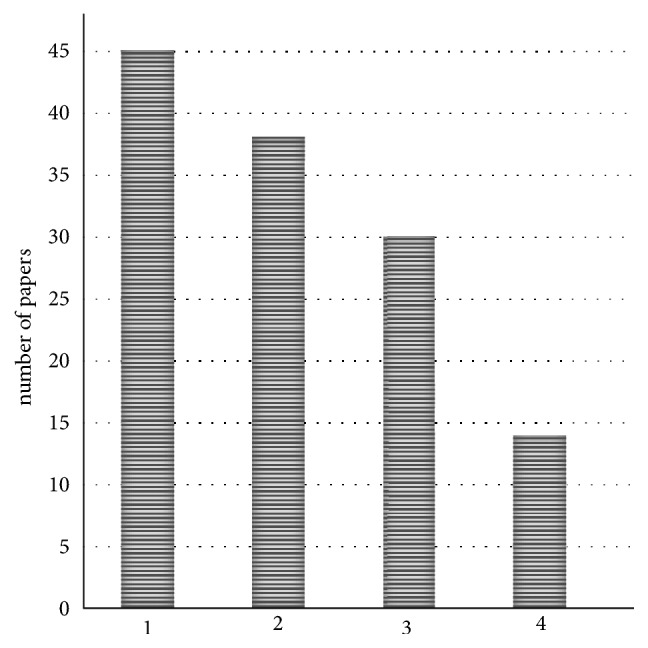
Number of papers for each subject studied for this manuscript. (1) Aging; (2) Alzheimer's disease; (3) Alzheimer's and aging; (4) other.

The most common subjects in the papers analyzed for this manuscript were aging and Alzheimer's disease (Figure 1).

The qui-square test indicated a significant difference [H_0_ accepted] considering the analysis in conjunction with the subjects for p<0,05.

The basic probability (P) is the number of the favorable cases/number of possible cases. For the theories of aging, the total number of subgroups studied here was 11 and 8 of them presented potential cases of generating AD; therefore,

(1) $$\begin{array}{c} P=\frac{number\;\;of\;\;favorable\;\;cases}{number\;\;of\;\;possible\;\;cases}=\frac{8}{11}=0.7272 \end{array}$$

## 4. Discussion

### 4.1. General Data on Aging

Aging is a process of random nature, with time-dependent and chronic-degenerative aspects that all individuals are necessarily subject to [39]. It is regulated by genetic and environmental factors [3, 7] and, in general, organs and tissues age at differing rates compared to the individual because they are used differently according to the individual's life style [40].

In general, the more an organ or tissue is used the more it will age [39], for instance, the ligaments of a football player compared to the normal and non-professional athlete. However, the situation seems to be different for the brain; more educational formation has been shown to prevent AD [41]. Putatively, the aging of tissues is associated with the decrease of cell renewal [42], which is not infinite; however, this is not the case for the brain and muscles where the cells have no mitotic capacity after differentiation [39].

Thus, the average number of cell mitosis in the organism is limited [43] and, during the aging process, a reduction of cellular regeneration capacity remarkably occurs [42].

Damage to DNA generates alterations in the mitosis cycles in organisms, in some cases, diminishing the cell count in tissues [44]. Therefore, the replacement of dead cells, caused by wound healing, atrophy, reduction of vascularization, and water content in tissues, which secondarily generate a decrease in weight and organ volume, remains in a deficit and may be slowed during healthy aging [3].

In fact, physiological aging generates a series of alterations in the organic and mental functioning in the organism, decreasing the capacity to maintain normal organic functions [45].

To explain the cited facts and others, various theories of aging were proposed. In general, and arbitrarily, they can be separated into 2 main categories, i.e., stochastic and non-stochastic [35].

### 4.2. Theories of Aging

Stochastic theories are associated with the loss of functions during aging due to the accumulation of aleatory lesions, in part, caused by environmental factors [46].

Stochastic theory presents the subgroups known as theory of somatic mutations, theory of error-catastrophe, theory of DNA reparation, theory of the breaking of chemical bonds, theory of advanced glycosylation, and theory of oxidative stress.

In summary, the theory of somatic mutations refers to that in which sublethal radiation diminishes over the course of the life time [21], increasing the probability of acquiring diseases [47], in a way, inter alia, due to biomolecular lesions. Thus, the normal radiation that people are exposed to during life diminishes life expectancy due to the destruction of biomolecules, mainly the DNA.

The error-catastrophe theory is associated with the perpetuation of protein synthesis errors which diminish the reliability of its production, creating aberrant and/or lethal proteins [35, 48] which could affect DNA replication and increase the probability of somatic mutations [49]. The reparation of DNA theory claims that the number of DNA replications determine the life span of a species [50] and could generate a higher probability of mutations within the DNA itself, therefore impairing proteins through the process of transcription.

The theory of the breaking of biomolecular bonds cites that the modification of proteins could generate the functional failure of cell metabolism [46], because of, and for instance, the increase of chemical bonds in DNA—collagen and elastin—resulting in a decline of the physiological processes during aging [51].

The glycosylation of proteins occurs from the cross-link between glucose and protein; collagen glycosylation has been the most studied and was associated with aging, according to the theory of advanced glycosylation [52, 53]. These cross-links are caused by a high concentration of glucose in the blood and tissues and results in functional deterioration [54], cases that are usual during aging.

Free radicals, or reactive oxygen metabolites (ROMs), are the basis of the theory of oxidative stress which is associated with the reactions of biomolecules with oxides and peroxides leading to the destruction of the biomolecules, causing many degenerative alterations associated with aging [36, 38, 55, 56]. Accordingly, aging is a consequence of the actions of ROMs on biomolecules, generating disease and death [38].

Despite this theory being backed by many reputable members within the scientific community [36], as well as laymen people, some data indicates that free radicals play no significant role in aging [57] and, recently, a theory of adaptive homeostasis was proposed as a more comprehensive explanation of the aging process [58].

The adaptive homeostasis theory considers anti-stress as a form of protection that maintains homeostasis within the organism; however, because this theory is recent and lacking in data, it will not be considered in the study of theories of aging for this paper (for more details, see [58]).

The environment and genetics act together on aging, according to non-stochastic theory [35] with subgroups such as theory of cellular aging or programmed senescence, theory of telomeres, theory of intrinsic mutagenesis, neuroendocrine theory, and immunological theory.

According to Hayflic [30] and the theory of programmed senescence, aging is based on genetic programming that controls cell development. The theory refers to the existence of an organic cell program that genetically determines the life span of each of the cells, which have finite capacities, and then the organism depletes and dies [40, 59, 60].

In fact, some genes are associated with diseases in the elderly, including some alleles of the apolipoprotein E which is also associated with AD [61]. Nevertheless, genes associated only with aging have not yet been found [35].

Aging is associated with the diminishment of cell repositioning, as cited above, that, in terms of chromosomes, is linked to the modification of telomeres, which are responsible for the integrity of chromosomes during cell division during life but are constantly depleted, diminishing the size of chromosomes [62]; this is the basis of the theory of telomeres. The existence of complete telomeres is dependent on the actions of the telomerase that decline after some time, in normal cells, resulting in the shortening of the telomeres and the genes disappearing from the region [63]. According to the telomeres theory, these genes may be associated with aging. These ideas were based on the fact that the cancerogenic cells have the telomerase working constantly, permitting the continuous division of cells [64].

According to another theory, the intrinsic mutagenesis, the longevity of an animal, depends of the reliability of genetic material in its replication, i.e., depends on a minor number of errors in DNA duplication that maintain the proper functioning of restorative enzymes [46, 49, 65]. Failure in DNA replication could generate mutagenesis, indicating a loss of functions in the organism and, thereby, causing the aging. In this case, proper protein production would be corrupted.

Some studies cite the influence of melatonin, which controls circadian rhythms, and associate it with aging [66]. This observation could play a part in the neuroendocrine theory that claims the decrease of many of the hormones of the hypothalamic-pituitary-adrenal axis causes problems with metabolism [46, 67–70], thereby causing the aging phenomenon.

According to immunologic theory, immunological responses decrease with aging, a fact observed in rodents and humans [46, 71], even with the creation of a self-antibody that diminishes the responses of the T-cells, generating low resistance to infections and diseases [72–74].

### 4.3. Some Characteristics of AD

Aging is associated with many factors, including biological, social, intellectual, economic, functional, and chronologic [21, 22]. Therefore, one theory alone would not cover all processes associated with aging. However, AD is strongly associated with genetic [8] and environmental components. The association of these factors could explain the various processes linked to aging and, even so, the behavior, economic, and social aspects will not be adequately elucidated. In this work, the molecular analysis was prioritized.

Long life increases the probability of contracting chronic diseases and generates physical incapacity [75]. Indeed, some dementias are directly associated with aging, particularly AD [10–13].

AD is associated with two types of prevalence: familiar and sporadic, which present the same clinical and nosologic signals [33]. The sporadic type is the most common and prevails from 65 years, while the familiar type can appear more early on [76]. Interestingly, in cases of trisomy of chromosome 21, AD can begin at around 30 years [77].

The neural deficit is progressive, generating mental deterioration with neurophysiological alterations [3], and these kinds of alterations could be used as a method for distinguishing AD from normal aging as a preclinical test [77], since advanced brain aging could be, or partially be, distinct from AD [78]. Indeed, the main problem is the overlapping between the brain features of those with AD and normal elderly people. Thus, minor aspects that differentiate normal aging from AD should be largely studied in attempt to differentiate dementia apart from normal aging.

The behavior and clinical development of AD seems to be associated with the senile plates formed by the ß-amyloid protein derived from the cleavage of amyloid precursor peptide (APP) [79–81], a process that occurs as a function of gene mutation [82] and, nowadays, Positron Emission Tomography has the ability to indicate the presence of ß-amyloid in the brain [83], as well as Magnetic Resonance Imaging [84]; however, this protein also exists in brains of normal aging people.

The amyloid protein induces the formation of abnormally phosphorylated* tau* protein generating neuron death [23, 85]. These neurofibrillary tangles are generated by the accumulation of paired helical filaments (PHF) whose main component is the abnormal phosphorylated* tau* protein [38]. The normal* tau* protein regulates the microtubules polymerization [86, 87], but* tau* seems to be associated with normal aging without generating AD [88]; however, traditional opinion has essentially discarded this perspective [89]. On the other hand, the* tau* could be found in different patterns in the brains of both younger and older people with AD [90]. There is more than one type of data that makes the diagnosis of AD difficult.

From the cited characteristics above, problems in AD are associated with protein alterations as a function of gene mutagenesis (ß-amyloid). Indeed, the gene of presenilin is present in chromosomes 1 and 14 [6], and in chromosome 19 the gene for apolipoprotein E is situated, all with defects associated with AD, and these mutations secondarily generate protein defects (*tau*), or at least the mutation of apolipoprotein E [91].

In addition to biomolecular problems, recent data indicate that AD is linked to epigenetic modifications [92] caused by the methylation of DNA [22].

Another protein called kallikrein 6 seems to be associated with amyloidogenic potential since it is found in relatively elevated quantities in cerebrospinal liquid in cases of AD [93] and, recently, aquaporin was associated with edema and microvascular alterations in the brains of those with AD [94, 95]; however, the diagnosis of AD using these proteins is not completely feasible today.

Neurotransmitters also suffer alterations in AD with a decrease in the production of acetylcholine [96, 97] and the inactivation of acetylcholinesterase is the basis for AD medicine. On the other hand, recent data indicate that norepinephrine in the locus coeruleus seems to be associated with the protection of neurons during aging [98].

In addition to genetic and protein alterations, factors such as aluminum intoxication, ROMs, and neurotoxic aminoacids are some well-known agents that could lead to the generation of AD [33] and disruption of blood-brain barrier was linked to the postmortem analysis of the brains of people with AD [99], as well as to the development of neuroinflammation [100]. Uncontrolled blood pressure could generate lesions in the small vessels [13] in the white matter with consequent gray matter atrophy [12, 101, 102].

### 4.4. Theories of Aging Associated with AD

In many articles, AD is directly associated with aging, however, not specifically with the theories of aging in general. Consequently, it is possible to associate both AD and aging with the theories of aging and some of the morphological, genetic, and biochemical aspects shown in these theories.

However, it is difficult to separate normal aging from such dementia as AD [11] since aging is a main risk factor for acquiring AD [103, 104]. Despite and due to limitations, these theories can be associated with aspects which are peculiar to AD, since it is very difficult distinguish normal aging from AD [105].

In general, all cited theories of aging of the stochastic type explain the etiology of AD in considering at least one possibility, except for the glycosylation theory. However, if it is not possible to affirm the action of the cross-link between glucose and proteins in neurons that affect AD, it is also not plausible to deny it.

In the theories of somatic mutations, DNA reparation could be associated with the mutagenesis of presenilin, ß-amyloid, and NFG expression genes; the theory of error-catastrophe could be linked to* tau* abnormal phosphorylation and the break of ligations with the abnormal fracture of ß-amyloid protein. Immunological theory is associated with the immunological effects that occur in aging as inflammatory processes [106] and activated microglia and astroglia was associated with AD [107].

Non-stochastic theories are more disconnected from the prevalence of AD. Programmed senescence and intrinsic mutagenesis could explain the gene alterations found in AD; however, the neuroendocrine theory could not generate sufficient information to justify AD etiology. Telomeres theory could be associated with depletion of genes linked to AD but there are no data on the localization of specific genes associated with AD located within telomeres. This does not discard this theory, regardless of whether or not is not possible to affirm its influence on this disease.

As far as considering the same probability of theories of aging to be correct, there are eleven in total, 6 stochastic and 5 non-stochastic. Therefore, in terms of basic probability, aging with AD is about 8/11 or 72,27%.

Of course, this probability does not reflect the reality of the number of people affected by AD in the world, because the 2018 estimate is 40 million people with AD [1], therefore, 40 million/7.6 billion (approximate number of people on Earth) resulting in an approximated number of 0,52%. Indeed, the failures of the theories of aging [108] and AD's unknown etiology do not permit an accurate and precise evaluation, in both one or the other, quantitative or qualitative analysis, concluding that the aging process is not completely known [109].

The theories of aging presented until now are more targeted towards a more catastrophic analysis, in relation to possibility of acquiring AD. Factors such as life expectancy could explain the fact that not so many people present AD; however, countries with lower life expectations present a higher prevalence of AD, i.e., developing countries [31]. Indeed, the error factors for a meticulous mathematics analysis are many and very difficult to control and to put into an equation.

An important point is that the prevalence of AD in the world is increasing exponentially (for a comprehensive review, see Kirova, Bays, Lagalwar [110], and Falco et al. [111]) putatively, due to declining mortality rate around almost all of the world. Another fact is that the prevalence of AD is increasing faster than human population growth [112]. Thus, suggesting that the increase of life expectancy, at least, is linked to the prevalence of AD in the world.

## 5. Conclusions

From the analysis performed above, it is not possible to distinguish AD from aging; however, aging is at least deeply linked to the prevalence of AD [113]. The problem could be in the separation of normal aging, i.e., healthy elderly people, from cases of AD [114]. Therefore, a question must be asked: is it possible to age without AD or, at least, some kind of senile dementia?

In other words, will AD appear definitively at some point in life and do the elderly people that do not present AD only not present it because they have not lived long enough?

As for whether or not the increase of mutations and metabolism errors sum to the fact that aging increases the disease's progression [115], the answer could be yes.

Thus, healthy aging entails only the avoidance of AD or dementia but not their prevention. It must be a controlled process that considers social, economic, behavioral, and mental factors; preventive aspects such as nutritional care could be associated with the avoidance of AD or the extension of the life span without AD [116]. Indeed, AD has a high social impact on the patient as well as on caregivers [31] and chronic psychosocial stress is a risk factor for AD [117]; i.e., aging and AD are associated with a genotype plus lifestyle [108].

Whether or not the genotype can be directly controlled, the life style can.

In a general sense, it seems that the theories of aging explain the origin of AD under superlative and catastrophic considerations and use more biomolecular data than social or behavioral data as the bases of analysis, which may be the problem.

According to references, the general idea is that AD is associated with aging in such a way that almost all people will present this disease; however, it is plausible to consider that the increase in life expectancy will generate a high prevalence of AD [118, 119]. Interestingly, it was calculated (p<0.05) that articles on AD published over recent years do not present similarity in terms of subjects and the prevailing association between Alzheimer and aging. Furthermore, from 2009 to 2014, the main topics covered were diagnosis, disease evolution, and treatment [120]. Therefore, there is a lack of papers considering the social and behavioral aspects of the prevention of AD.

Thus, it seems that healthy aging is associated with the control of social, economic, metabolic [121], behavioral, and mental activities; however, familiar dementia, gender, depression, head trauma, smoking, high blood pressure, heart diseases, and stroke do not seem to be associated with AD, according to some studies (for more details, see Lindsay et al. [122]), therefore, generating conflict regarding these aspects cited in many papers as imperative to the acquiring of AD.

In this article, the social aspects were not discussed because the focus was on AD and the theories of aging; however, the prevalence of AD associated with patterns of life must be researched more thoroughly with an increase in the number of subjects studied because AD is a disease that there is no cure for. Therefore, prevention is the best process to avoid it, especially considering AD as a public health problem [1, 31].
